# Supplementary figures and images for: Human cytomegalovirus-IE2 suppresses antigen presentation of macrophage through the IL10/STAT3 signalling pathway in transgenic mouse
Source: PLoS One. 2025 May 5;20(5):e0322334. doi: 10.1371/journal.pone.0322334 (PMC12052161; doi:10.1371/journal.pone.0322334)

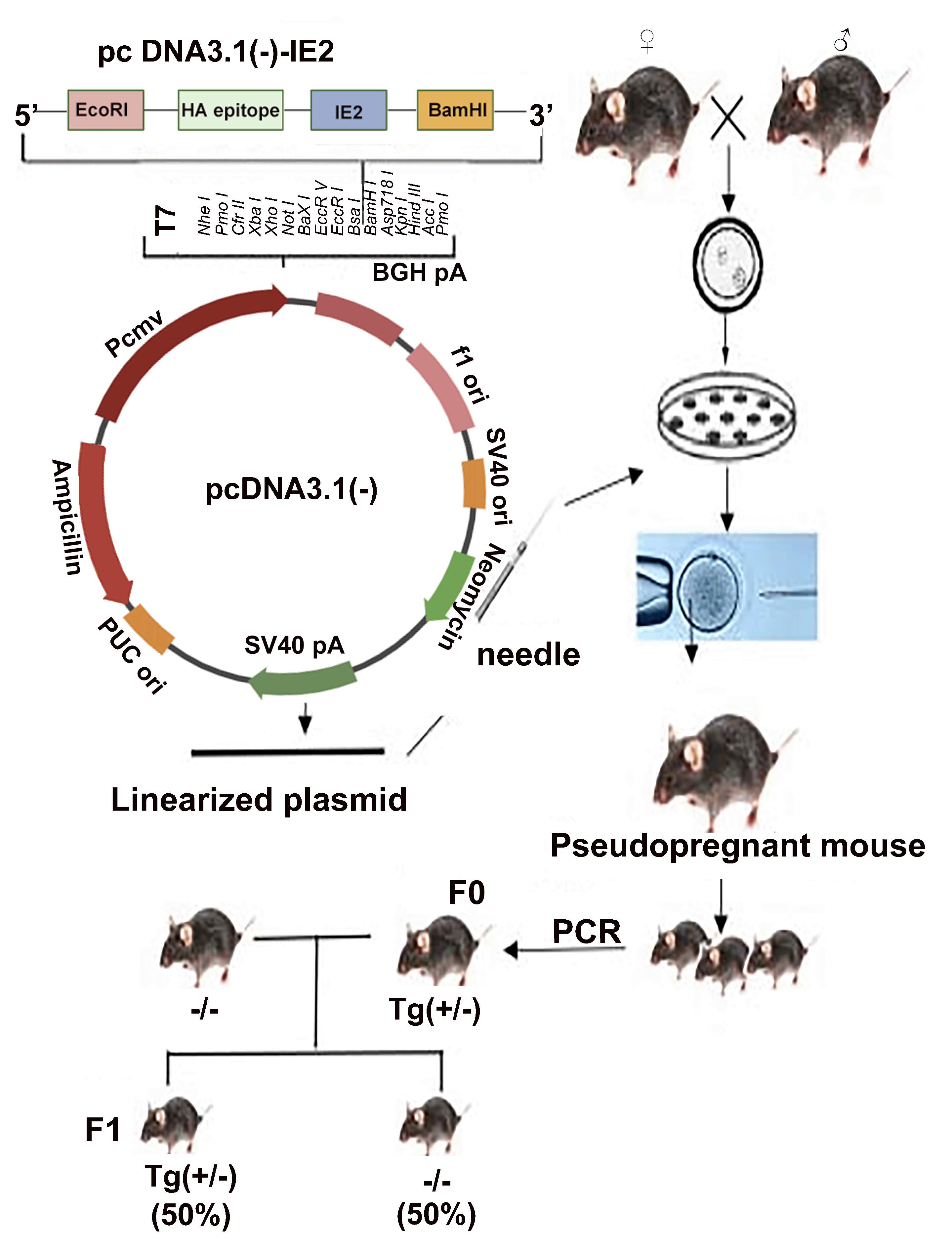

Supplement: S1 Fig — (TIF) [file pone.0322334.s001.tif]

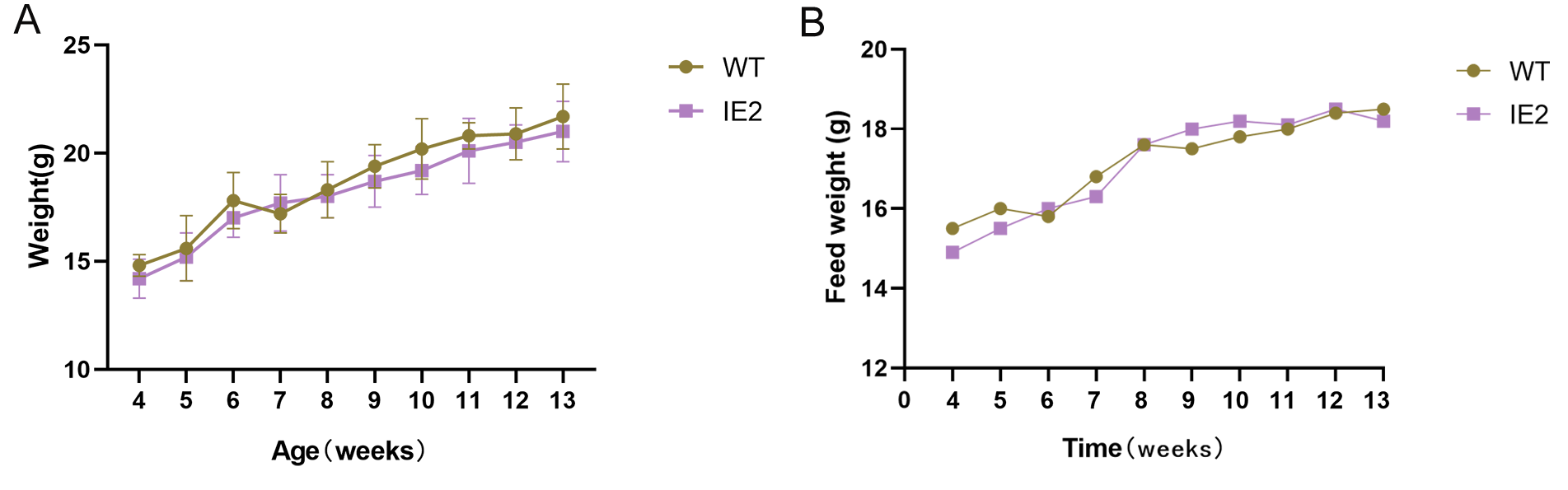

Supplement: S3 Fig — (A) Body weight changes in different groups. (B) Food intake changes in different groups. (TIF) [file pone.0322334.s003.tif]

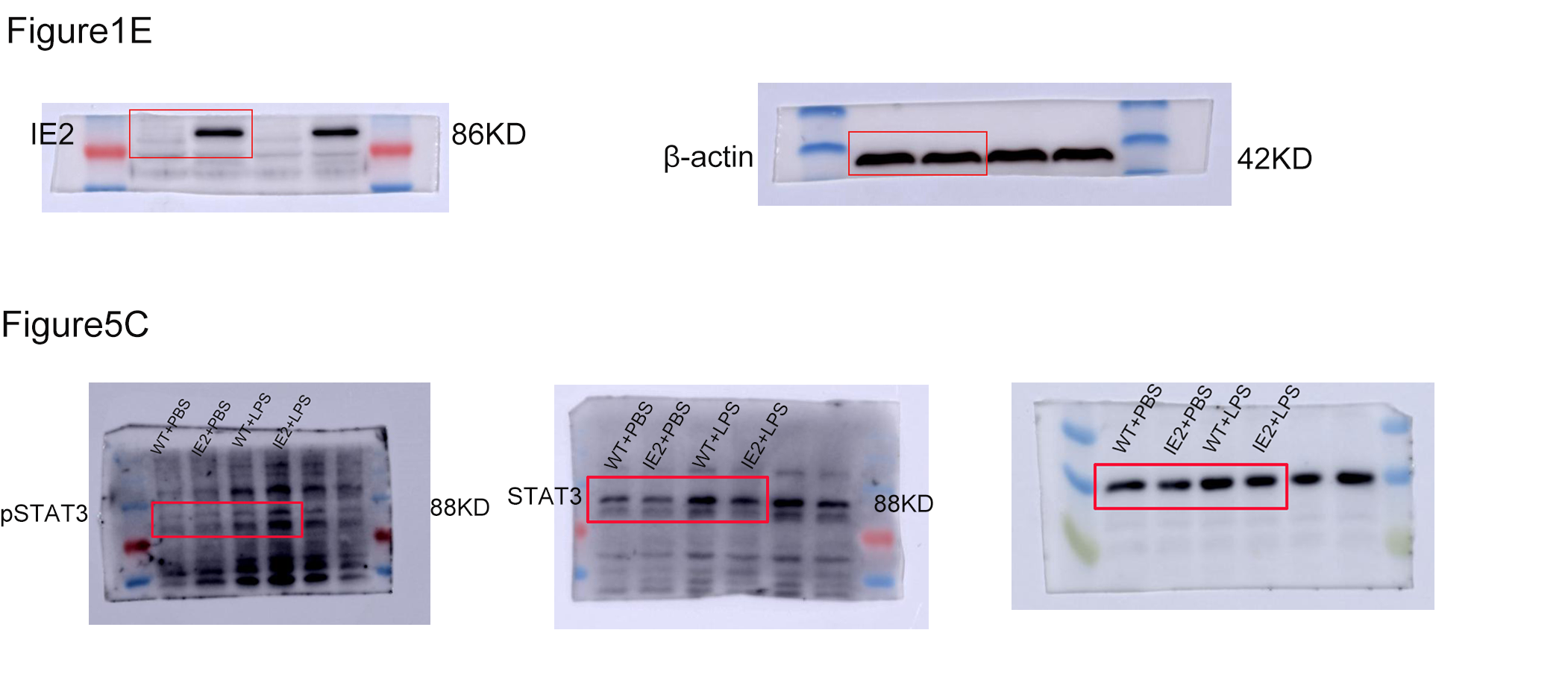

Supplement: S4 Raw Images — (TIF) [file pone.0322334.s004.tif]
