# Supplementary material for: Human cytomegalovirus-IE2 suppresses antigen presentation of macrophage through the IL10/STAT3 signalling pathway in transgenic mouse
Source: PLoS One. 2025 May 5;20(5):e0322334. doi: 10.1371/journal.pone.0322334 (PMC12052161; doi:10.1371/journal.pone.0322334)
Supplement: Attachment — Submitted filename: Point by Point Response.docx [file pone.0322334.s007.docx]

Dear Editors and Reviewers,

Thanks very much for taking your time to review this manuscript (manuscript number: PONE-D-24-54197R1). I really appreciate all your comments and suggestions! These suggestions have enabled us to improve our work. Based on the instructions provided in your letter, we revised the original and marked the modified part by red in revised manuscript (with changes marked). In addition, in order to make the expression of the manuscript more standardized, we have carried out language editing, which may lead to the unmarked parts in the text are not completely consistent with the original manuscript, but the content is the same. Please find my itemized the point to point responses in below. In case of further queries, we are happy to clarify any details and look forward to your reply.

Thanks again!

Sincerely,

Xianjuan Zhang

Corresponding author: Bin Wang；Yunyang Wang

E-mail: [wangbin532@126.com](mailto:wangbin532@126.com)；wangyy_09@outlook.com.

**Journal Requirements:**

**1.Thank you for stating the following financial disclosure: 
This research was funded by Shandong Provincial Science and technology Foundation (grant no. 2019JZZY011009), Qingdao Municipal Science and technology Foundation (grant no. 20-2-3-4-** **nsh), National Key Research and Development Program of China (grant no.2018YFA0900802), Shandong Provincial Natural Science Foundation (grant no. ZR2021QH254).
Please state what role the funders took in the study.  If the funders had no role, please state: "The funders had no role in study design, data collection and analysis, decision to publish, or preparation of the manuscript." If this statement is not correct you must amend it as needed. Please include this amended Role of Funder statement in your cover letter; we will change the online submission form on your behalf.**
**Response:** Thank you for your attention to our financial disclosure statement. As requested, we hereby confirm the role of the funders in the study as follows: The funders had no role in study design, data collection and analysis, decision to publish, or preparation of the manuscript. We will include this statement in our cover letter for submission.

**2. PLOS ONE now requires that authors provide the original uncropped and unadjusted images underlying all blot or gel results reported in a submission’s figures or Supporting Information files. This policy and the journal’s other requirements for blot/gel reporting and figure preparation are described in detail at https://journals.plos.org/plosone/s/figures#loc-blot-and-gel-reporting-requirements and https://journals.plos.org/plosone/s/figures#loc-preparing-figures-from-image-files. When you submit your revised manuscript, please ensure that your figures adhere fully to these guidelines and provide the original underlying images for all blot or gel data reported in your submission. See the following link for instructions on providing the original image data: https://journals.plos.org/plosone/s/figures#loc-original-images-for-blots-and-gels.**

**In your cover letter, please note whether your blot/gel image data are in Supporting Information or posted at a public data repository, provide the repository URL if relevant, and provide specific details as to which raw blot/gel images, if any, are not available. Email us at plosone@plos.org if you have any questions.**
**Response:** Thank you for your feedback and for bringing PLOS ONE’s updated requirements for blot and gel reporting to our attention. We have carefully reviewed the journal’s guidelines at the provided links and ensured that our figures and supporting materials fully comply with these requirements. We have prepared the original uncropped and unadjusted images underlying all blot and gel results reported in the Supporting Information files accompanying our revised submission. In our cover letter, we have included the following statement to confirm compliance with PLOS ONE’s requirements: The original uncropped and unadjusted images underlying all blot and gel results reported in this manuscript are provided in the Supporting Information.

**3. "We note that your Data Availability Statement is currently as follows: [All relevant data are within the manuscript and its Supporting Information files.]
Please confirm at this time whether or not your submission contains all raw data required to replicate the results of your study. Authors must share the “minimal data set” for their submission. PLOS defines the minimal data set to consist of the data required to replicate all study findings reported in the article, as well as related metadata and methods (https://journals.plos.org/plosone/s/data-availability#loc-minimal-data-set-definition). 
For example, authors should submit the following data:
- The values behind the means, standard deviations and other measures reported;
- The values used to build graphs;
- The points extracted from images for analysis.
Authors do not need to submit their entire data set if only a portion of the data was used in the reported study.
If your submission does not contain these data, please either upload them as Supporting Information files or deposit them to a stable, public repository and provide us with the relevant URLs, DOIs, or accession numbers. For a list of recommended repositories, please see https://journals.plos.org/plosone/s/recommended-repositories. 
If there are ethical or legal restrictions on sharing a deidentified data set, please explain them in detail (e.g., data contain potentially sensitive information, data are owned by a thirdparty organization, etc.) and who has imposed them (e.g., an ethics committee). Please also provide contact information for a data access committee, ethics committee, or other institutional body to which data requests may be sent. If data are owned by a third party, please indicate how others may request data access."**
**Response:** Thank you for your note regarding our Data Availability Statement. We confirm that our submission includes the minimal data set required to replicate the results of our study, as defined by PLOS ONE. All raw data, including the values behind means, standard deviations, and other measures, the data used to build graphs, and the points extracted from images for analysis, are provided within the Supporting Information files accompanying our manuscript.

**4. “Thank you for responding to our previous query.
please provide additional information regarding the experiments involving animals and ensure you have included details on methods of sacrifice. We hope you understand the reasons behind this request and look forward to hearing from you."**

**Thank you for your follow-up query regarding the experiments involving animals. We appreciate the importance of providing detailed information to ensure transparency and reproducibility in our research.**

**Response:** In response to your request, we have updated the **Materials and Methods**

 Section (lines 58-68) of our manuscript to include additional details regarding the animal experiments, specifically the methods of sacrifice.

**Comments to the Author:**

**Reviewer #1:**

**In this study, Dr. Zhang and collaborators take advantage of a transgenic mouse model stably expressing the HCMV IE2 protein to prove that the long-term IE2 expression has a major negative impact on immune activation. Although some of the presented results are suggestive, the primary issue I can envisage is the lack of supporting experiments using human cells. These should be easily obtained with primary human macrophages transduced with a plasmid carrying the IE2 gene and with HCMV strains deleted for this gene. The author should explain why they did not pursue these approaches.
In general, the findings are poorly explained, and the majority of the experimental details are vague. Finally, the English language need accurate revision.**

**Response:** Thank you for your valuable comments. We appreciate the opportunity to address your concerns. Our study focuses on the mechanisms of IE2 in a mouse model, which provides a systematic biological context for understanding its function. Additionally, mouse models are commonly used in immunology, and their macrophages share similarities with human macrophages. Thus, our findings can offer insights into HCMV's role in humans. We intend to investigate IE2's role in primary human macrophages and conduct experiments with HCMV strains lacking the IE2 gene in future studies. We hope this clarifies our approach and addresses your concerns. Thank you again for your feedback, which has helped us improve our manuscript.

**Specific details:**
**1. the construct for transgene generation is poorly described.**

**Response:** Thank you for your valuable feedback regarding the description of the transgenic construct. We appreciate your suggestion and have expanded the M&M section to provide a more detailed account of the construction process (lines 69-73). We believe this additional information will enhance the clarity and comprehensiveness of our study.

**2. in Fig 2 and Fig 3: the authors should mention in the text or at least in the legend what T stands for (in WT+T and IE2+T).**

**Response:** Thank you very much for your insightful comments and suggestions regarding our manuscript. We greatly appreciate your attention to detail, which has helped us improve the clarity of our work. In response to your observation about Figures 2 and 3, we have updated the text and figure legends to explicitly define what "T" represents in the contexts of WT+T and IE2+T (lines 190-191). We believe this clarification enhances the reader's understanding of our findings.

**3. for Fig 3: a few details are provided in both the text and the legends. Please amend.**

**Response:** Thank you for your thorough review and valuable feedback on our manuscript. Regarding your comments about the details provided in Fig 3, we have carefully revised both the text and the figure legend (lines 205-219).

**4. Fig 3C: please change “E Coil” to “E. coli”**

**Response:** Thank you for your careful review and for pointing out the error in Figure 3C. We have corrected “E. coil” to “E. coli” as you suggested. Correction has been made in the revised version.

**5. Fig 3E, F: how much MCP-1 was used for the transwell experiment? Please include details in the main text and/or in the** **M&M section**

**Response:** Thank you for your valuable feedback regarding the transwell experiments presented in Figures 3E and 3F. We appreciate your suggestion to include more details about the concentration of MCP-1 used in these experiments. We have now added the specific concentration of MCP-1 to the M&M section of the manuscript for clarity (lines 113-116). Additionally, we have referenced this information in the main text to ensure that readers can easily find it (lines 190-191).

**6. In numerous places throughout the text, the MCP-1 acronym is wrongly reported as Mcp-1.**

**Response:** Thank you for your valuable feedback regarding our manuscript. We appreciate your attention to detail and the time you have taken to review our work. In response to your comment about the abbreviation for MCP-1, we acknowledge that it was incorrectly reported as "Mcp-1" in several places throughout the manuscript. We have carefully reviewed the text and corrected all instances to the proper format "MCP-1" to ensure consistency and accuracy.

**7.** **M&M and legends are poorly detailed, with some inaccuracies (i.e., line 197: The Transwell® system WERE used)**

**Response:** Thank you for your constructive feedback regarding the M&M section, as well as the legends. We have revised the M&M and legends section to provide more comprehensive details and have corrected the inaccuracies you pointed out. Additionally, we have ensured that all other relevant details are clearly presented and accurate.

**Reviewer #2**

**Major issues:**

**1. This is a new transgenic mouse model. While Figure 1 adequately shows where in the genome IE2 was knocked-in and identified mice with the knock-in by PCR, its important to provide more general information about the health of the mice–do they live as long as WT counterparts? Do they have any unexpected abnormalities? I am sure the authors have noted these things in the development of the transgenic mice – as a reader, I want to know these things to aid in my interpretation of the rest of the paper.**

**Response:** Thank you for your valuable feedback regarding our newly developed transgenic mouse model. We appreciate your inquiry into any unexpected abnormalities observed during the development of these mice, as this information is indeed important for interpreting our findings. In our study, we have observed that the transgenic mice develop learning and memory impairments and metabolic disturbances after 12 weeks of age. However, it is important to note that the mice used in our experiments were 6-8 weeks old, a developmental stage at which we have not observed any significant impacts on weight or dietary intake. Therefore, we believe that the effects of IE2 on immune responses can be accurately assessed without the confounding factors associated with the later-stage abnormalities. We have added a discussion in the revised manuscript to clarify these observations and their implications for our study (lines 322-339).

**2. Line 52, p.3: “…mouse model simulates the expression process of viral proteins after HCMV infection in vivo to a certain extent.” – there is no data provided for this…? To WHAT extent? It is important to show more characterization of this mouse model, especially when claiming that it recapitulates the expression process. HCMV expresses genes in the typical cascade of IE->E->L, and this model relies on long-term sustained expression of IE2. How is this simulating the “normal” expression of IE2? Data on this would be extremely useful to aid readers in interpreting the reliability of extrapolating data from this study to what we think is going in in humans/human cells.**

**Response:** Thank you for your thorough review of our manuscript and for your valuable feedback regarding the mouse model. We appreciate your concerns about the need for data supporting the extent to which our model simulates the expression of viral proteins following HCMV infection. In the revised manuscript, we have included a more detailed description of the characteristics of the mouse model, and added the study's limitations in the discussion section. Our study is based on the construction of an IE2 transgenic mouse, which relies on the prolonged expression of IE2 to simulate its effects on macrophages following HCMV infection. While we acknowledge the limitation of not being able to quantitatively measure IE2 expression levels directly, we emphasize the significance of this model in understanding the role of IE2 in modulating immune responses.

**Minor issues:**

**1. Line 40 p.2: “macrophages are the first targets during HCMV infection.” – it is often said that epithelial, endothelial, or fibroblast cells are the first cells infected when a host encounters the virus, and that trafficking from these outer tissues to the endothelium allows the virus to encounter cells like macrophages. I definitely agree they are important for viral persistence and spread, but I would not definitively declare macrophages as the first target cells of HCMV.**

**Response:** Thank you for your insightful comment. We appreciate your perspective on the initial target cells during HCMV infection. While our previous statement that macrophages are the first targets of HCMV infection was based on [1]. We acknowledge that the literature also supports epithelial, endothelial, and fibroblast cells as primary targets. To avoid ambiguity, we have revised the text to clarify that while macrophages play a significant role in the persistence and dissemination of HCMV. We have made this adjustment to ensure a more accurate representation of the infection dynamics in our manuscript (lines 39-40).

1. Bayer C, Varani S, Wang L, Walther P, Zhou S, Straschewski S, Bachem M, Söderberg-Naucler C, Mertens T, Frascaroli G. Human cytomegalovirus infection of M1 and M2 macrophages triggers inflammation and autologous T-cell proliferation. J Virol. 2013 Jan;87(1):67-79. doi: 10.1128/JVI.01585-12. Epub 2012 Oct 10. PMID: 23055571; PMCID: PMC3536399.

**2. Entire introduction – I would like more specific information included in the introduction, describing a bit more specifically what the cited studies showed. For example, line 43 “Previous studies have shown that HCMV infection mediates immune escape by regulating macrophage activity.” – how does it regulate it? What was shown? What part of macrophage activity was regulated? Was it IE2 or something else? What kinetic class of viral gene (if known) was implicated in regulating macrophage activity? What models were used? Etc.**

**Response:** Thank you for your insightful comments regarding the introduction. We agree that providing more specific details regarding the mechanisms of HCMV-mediated immune evasion through macrophage modulation will strengthen the manuscript (lines 42-44).

**3. Fig. 1A – This is a nice map of the insertion, but it could use some more detail. Under what promoter was IE2 expression placed? The font is also quite small – readability could be improved by altering font sizes.**

**Response:** Thank you for your comments. We have increased the font size in Figure 1A to improve readability. We have now clarified that IE2 expression is driven by both CMV and T7 promoters, as indicated in the revised figure.

**4. Lines 148-149: “indicating that the UL122 gene insertion does not affect the expression of neighboring genes.” – The assumption is the gene was inserted far enough away from neighboring genes to not alter expression. Including control RTqPCR or Western blots for Fam47c and Cfap47-205 in WT vs IE2 mice would strengthen this argument.**

**Response:** Thank you for your raising this important point. While we believe the distance of the UL122 insertion from Fam47c and Cfap47-205 minimizes the risk of transcriptional interference, we acknowledge the value of direct evidence. We have added RT-qPCR data for these genes in both WT and IE2 mice (Figure 1C).

**5. Line 158: “In the spleen, Mos were stained with CD11b+” – were cells isolated from the spleen specifically and then stained? Or is this some in vivo staining?**

**Response:** Thank you for your insightful question. In our study, we performed cell isolation from the spleen prior to the staining process. The CD11b+ cells were specifically separated from the splenic tissue and then subjected to staining. This approach allows for a more accurate analysis of the specific cell populations of interest. We appreciate your attention to detail and hope this clarification addresses your concern.

**6. Throughout (example line 171) – specify that it is continued/sustained IE2 expression resulting in your phenotypes.**

**Response:** Thank you for raising this important point. We agree that clarifying this aspect is crucial to interpreting our results. The IE2 transgene was introduced into the mouse genome via pronuclear microinjection, resulting in constitutive IE2 expression in mouse. Thus, our results suggested that the sustained IE2 expression may lead to changes in immune cell phenotype.  We have made additions in the revised manuscript.

**7. Figure 2B: the data are compelling on their own, but my first thought was to calculate in my head the fold changes relative to untreated for each mouse. It would be helpful to include in the figure the same data plotted relative to untreated (as a fold change).**

**Response:** Thank you for your suggestion to present the data as fold change relative to the untreated control. We agree that this will enhance the clarity and interpretability of Figure 2B. We have therefore added a new panel to Figure 2B displaying the fold change for each mouse compared to its corresponding untreated control.

**8. Figure 3A-B: as a non-immunologist, it took awhile to parse out which data meant M1 vs M2. This could easily be fixed by adding labels to the figure, and strengthen the figure by making it more readable to people outside the field.**

**Response:** Thank you for your comment regarding the clarity of Figures 3A and 3B. We agree that the distinction between M1 and M2 macrophage populations was not immediately apparent to a non-immunologist. To address this, we have revised the figures. Specifically, we have added a detailed the markers used to identify M1 and M2 macrophages, iNOS for M1, CD206 for M2, and included a brief explanatory paragraph in the figure legend providing a concise overview of M1 and M2 macrophage polarization (lines 210-214). These changes enhance the interpretability of the data and make the figures more accessible to a wider readership.

**9. Lines 185-188: The experiment could use more explanation (again mainly for those readers who don’t frequently do phagocytosis assays) – where does the GFP come from? What do the images tell us specifically?**

**Response:** Thank you for your suggestion to improve the clarity of the phagocytosis assay description. To enhance understanding for readers unfamiliar with this technique, we have expanded the methods section with a more detailed explanation (lines 118-129). Specifically, Labeling of E.coli with FITC tags for the detection of phagocytosis in macrophages(1,2). This allows for easy visualization and quantification of phagocytosis. Figures3C and D present representative microscopy images showing FITC-positive macrophages, and a graph depicting the quantitative analysis of phagocytic activity, expressed as the percentage of FITC-positive macrophages. We believe these additions significantly improve the clarity and accessibility of this section.

（1）Li Z, Zhang S, Liu Q. Vitellogenin functions as a multivalent pattern recognition receptor with an opsonic activity. PLoS One. 2008 Apr 9;3(4):e1940. doi: 10.1371/journal.pone.0001940. PMID: 18398466; PMCID: PMC2277463.

（2）Qiao H, Wang Y, Zhang X, Lu R, Niu J, Nan F, Ke D, Zeng Z, Wang Y, Wang B. Cross-species opsonic activity of zebrafish fish-egg lectin on mouse macrophages. Dev Comp Immunol. 2022 Apr;129:104332. doi: 10.1016/j.dci.2021.104332. Epub 2021 Dec 12. PMID: 34910945.

**10. Lines 189-192: this simple inclusion of “the chemokine MCP-1 is known to induce migration” is a great example of the minor addition of explanation (needed for the phagocytosis assay) really making a difference for my understanding. This was great!**

**Response:** Thank you for your positive feedback regarding the inclusion of the explanation about MCP-1's role in inducing migration. We strive to provide clear and informative explanations to ensure that our findings are accessible to all readers, and your acknowledgment of this effort is greatly appreciated.

**11. Fig 3E legend (line 197) – this could use more detail.**

**Response:** Thank you for your thorough review and valuable feedback on our manuscript. Regarding your comment about the need for more details in the legend for Figure 3E, we will revise the legend to provide clearer and more comprehensive information for better understanding (lines 216-217).

**12. Effect of IE2 on T cells (lines 201-213): This section could use a bit more detailed explanation of the question you’re asking, what you measured by flow cytometry, and what it means for both CD4 helper T cells AND CD8 cytotoxic T cells (line 213 seems specifically focused on CD4 – what about CD8? What are the implications there?)**

**Response:** Thank you for your valuable feedback regarding our manuscript. CD8 T cells are stimulated to differentiate into cytotoxic T cells for killing infected cells.  Activation of Th1 cells can also enhance the killing function of CD8 T cells, whereas Th2 cells inhibit the activity of macrophages and Th1 cells. Regarding the use of flow cytometry, the relative proportions and functional states of CD4 T cells and CD8 T cells under different treatment conditions were measured mainly by flow cytometry. In our experiments, we examined the cytokine expression in CD4 T cells, including IFN-γ (Th1 cytokine) and IL-4 (Th2 cytokine); and the cytokine expression in CD8 T cells, including IFN-γ and TNF-α. The results showed that IE2 significantly promoted the Th2-type immune response by decreasing the IFN-γ expression of CD4+ T cells while increasing their IL-4 expression. In contrast, CD8+ T cells showed significantly lower IFN-γ and TNF-α expression in the IE2+LPS group, suggesting an inhibitory effect of IE2 on cytotoxic T cells. These changes suggest that IE2 modulates T cell-mediated immune responses by promoting Th2-type immune responses and suppressing Th1-type immune responses. We made more detailed changes in this results section (lines 220-238).

**13. Figure 4: As mentioned for figure 2B (point 7 above), it would be useful to plot fold changes relative to the PBS control for each mouse in addition to the data presented. This would clearly show if there’s a specific change in activation as opposed to baseline changes overall in the IE2 mouse (this would also be interesting on its own!)**

**Response:** Thank you for your insightful feedback regarding Figure 4. We appreciate your suggestion to plot fold changes relative to the PBS control for each mouse. We agree that this additional analysis would provide valuable information on the specific changes in activation in the IE2 mouse model compared to baseline changes. We have now included the fold change data in the revised figure.

**14. Figure 5: Again, this looks at the changes in IE2 vs WT in LPS-stimulated cells; however, it would be interesting to also include expression of these MHC markers or cytokines at baseline (without LPS) for IE2 and WT mice. The fold change compared to baseline would again be helpful. Same for the STAT3 Western blots.**

**Response:** Thank you for your insightful suggestion. We agree that including baseline expression levels of MHC markers, cytokines (specify which ones), and STAT3 in both IE2 and WT mice would provide a more comprehensive analysis of the LPS-induced effects. We have performed the necessary experiments and included the baseline data, along with the fold change calculations relative to baseline, in Figure 5 (revised).

**15. Line 263: what is “E” gene product?**

**Response:** Thank you for your inquiry regarding the "E" gene product. In permissively infected cells, HCMV gene expression is regulated in a cascade fashion characteristic for herpes viruses. Gene can be categorized into three types: immediate early (IE), early (E), and late (L). HCMV proteins encoded by genes expressed in the immediate-early (IE) phase of infection control the activation of early (E) phase genes (1). E gene product is E protein. E proteins are required for viral DNA synthesis, which is followed by the synthesis of structural proteins during the late phase of infection (2). We will ensure that this information is clearly articulated in the revised version of the manuscript to enhance understanding (287-293).

(1) Shang Z, Li X. Human cytomegalovirus: pathogenesis, prevention, and treatment. Mol Biomed. 2024 Nov 25;5(1):61. doi: 10.1186/s43556-024-00226-7. PMID: 39585514; PMCID: PMC11589059.

(2) Del Val M, Hengel H, Häcker H, Hartlaub U, Ruppert T, Lucin P, Koszinowski UH. Cytomegalovirus prevents antigen presentation by blocking the transport of peptide-loaded major histocompatibility complex class I molecules into the medial-Golgi compartment. J Exp Med. 1992 Sep 1;176(3):729-38. doi: 10.1084/jem.176.3.729. PMID: 1324970; PMCID: PMC2119349.

**16. Line 293: This study revealed a correlation between IE2 expression and changes in IL-10/STAT3, but the mechanism feels lacking in data. This conclusion would be more fully supported if trans-complementing studies or something could be done – I am not sure whether that is meant to be in the scope of this paper or not. Thus, you could simply say “our study SUGGESTS a mechanism” instead of “our study REVEALS”.**

**Response:** Thank you for your valuable feedback regarding our manuscript. We appreciate your observation about the correlation between IE2 expression and changes in IL-10/STAT3, and we acknowledge the need for more data to support the proposed mechanism. We will revise the wording in the manuscript to reflect your suggestion and state that "our study suggests a mechanism" rather than "our study reveals a mechanism." We agree that this change will provide a more accurate representation of our findings (lines 340-341).

**17. The last sentence regarding vaccine development is not necessary. Final thoughts: these data suggest quite a cool link between IE2, STAT3/IL10, and macrophage activity. Looking forward to seeing more in depth follow up studies in the future.**

**Response:** Thank you for your thoughtful review and feedback on our manuscript. We appreciate your perspective on the last sentence and will consider making adjustments in the revised version to enhance clarity and conciseness. We are also grateful for your insights into the intriguing connections between IE2, STAT3/IL10, and macrophage activity. Like you, we look forward to conducting further research to explore these mechanisms in greater depth.
