# Supplementary material for: Human cytomegalovirus-IE2 suppresses antigen presentation of macrophage through the IL10/STAT3 signalling pathway in transgenic mouse
Source: PLoS One. 2025 May 5;20(5):e0322334. doi: 10.1371/journal.pone.0322334 (PMC12052161; doi:10.1371/journal.pone.0322334)
Supplement: Attachment — Submitted filename: Point_by_Point_Response_auresp_2.docx [file pone.0322334.s008.docx]

Dear Editors and Reviewers,

Thank you very much for taking the time to review our manuscript (Manuscript Number: PONE-D-24-54197R2). We greatly appreciate your insightful comments and constructive suggestions, which have significantly enhanced the quality of our work.

In response to your feedback, we have carefully revised the manuscript accordingly. All modifications have been highlighted in red in the revised version. Additionally, to ensure the manuscript meets high linguistic standards, we have conducted professional language editing. While this may result in some unmarked textual variations from the original manuscript, we assure you that the core content remains unchanged.

Please find below our point-by-point responses to your comments. Should you have any further questions or require additional clarification, we would be more than happy to provide further details. We look forward to hearing from you.

Thanks again!

Sincerely,

Xianjuan Zhang

Corresponding author: Bin Wang；Yunyang Wang

E-mail: [wangbin532@126.com](mailto:wangbin532@126.com)；wangyy_09@outlook.com.

**Comments to the Author:**

**Reviewer #1:**

**1. The authors have added some details about the generation of the transgene model, both in the M&M section and in the new Fig. S1. However, in the M&M the new paragraph sounds: “A pAV.Ex1d-CMV-IE2 vector containing a cDNA fragment of the IE2 gene was constructed, and the vector was injected into…”. No details are provided about the origin of the viral gene: where does this gene come from? What strategy did they employ for its amplification and cloning?**

**Response:** Thank you very much for your insightful comments and suggestions regarding our manuscript. We greatly appreciate your attention to detail, which has helped us improve the clarity of our work. In response to your suggestion, we have now provided comprehensive details about the viral gene origin and cloning strategy in the revised M&M section (lines 73-87).

**2. Furthermore, the quality of figure S1 is poor, with some black squares partially covering the image and a scheme from the company's website that I'm not convinced can be used in this context.**

**Response:** Thank you for bringing these issues with Figure S1 to our attention. We sincerely apologize for the poor image quality. We have completely revised Figure S1 in the updated manuscript to address these concerns.

**3. Finally, some more editing of the English language is required before acceptance.**

**Response:** Thank you for pointing out the need for additional language editing. We sincerely appreciate your careful reading of our manuscript and your valuable suggestion. In order to improve the English language quality throughout the manuscript, the manuscript has been professionally edited by Elsevier Language Editing Services (Order reference: ASLESTD1076952). We believe these professional edits have significantly improved the clarity and readability of our manuscript while maintaining its scientific integrity.

**Reviewer #2**

**The authors have adequately addressed reviewer concerns, which have strengthened the paper to be of higher quality for publication.**

**Response:** We are sincerely grateful for your positive feedback and for recognizing our efforts. We greatly appreciate the time and expertise you have dedicated to improving our manuscript. We are honored by your assessment that our manuscript now meets the journal's publication standards. Thank you again for your valuable guidance, which has undoubtedly strengthened our work.
